# Randomized community trial to assess nutritional, socioeconomic, and health outcomes of a food forest initiative in Santa Elena Province, Ecuador: a study protocol

**DOI:** 10.3389/fpubh.2024.1420310

**Published:** 2024-12-24

**Authors:** Marco Faytong-Haro, Alonso Quijano-Ruiz, Daniel Sanchez, Patricio Alvarez-Munoz, Stephanie Gallegos-Caamaño, Victor Yambay-Delgado, Karina Checa, María José Delgado-Rendón, Andrea Angulo-Prado

**Affiliations:** ^1^Facultad de Ciencias de la Salud, Universidad Espíritu Santo, Samborondon, Ecuador; ^2^Research Department, Ecuadorian Development Research Lab, Daule, Ecuador; ^3^Department of Economics, Simon Fraser University, Burnaby, BC, Canada; ^4^Department of Statistics, University of Salamanca, Salamanca, Spain; ^5^Facultad de Ciencias Sociales, Educación Comercial y Derecho, Universidad Estatal de Milagro, Milagro, Ecuador; ^6^Community Engagement Department, CECAPROF Corporación Ecuatoriana De Capacitación, Guayaquil, Ecuador; ^7^Centro de Investigación, Instituto Superior Tecnológico Argos, Guayaquil, Ecuador

**Keywords:** food forest, nutrition, rural area, Ecuador, intervention, pre-post study, three time slots

## Abstract

Malnutrition is an escalating concern in low-and-middle-income countries (LMICs), including Ecuador, particularly within rural settings. To address this issue, food forests emerge as a promising intervention. This research protocol outlines a controlled intervention in the province of Santa Elena, aiming to evaluate the efficacy of a food forest in enhancing nutritional outcomes, with potential implications for broader replication. The study will be conducted in the Colonche Parish of Santa Elena Canton, where one commune will be randomly selected to receive the food forest intervention. In contrast, another similarly characterized commune, also randomly selected through cluster-based sampling, will serve as a control group, receiving no intervention. This randomized, comparative approach will enable a more precise assessment of the food forest's impact. Data collection will occur at three intervals: baseline, 6 months, and 12 months post-intervention. Comprehensive questionnaires will be employed to measure the food forest's influence on the communities' nutritional, economic, and health metrics, distinguishing between the intervention and control communes to elucidate the intervention's specific effects.

## 1 Introduction

Malnutrition is a severe global challenge, particularly in low- and middle-income countries (LMICs), where it causes ~3.1 million child deaths annually, representing nearly 45% of all child fatalities (1–5). Poor nutrition in the early years of life, especially in vulnerable populations, can lead to long-term adverse effects such as impaired cognitive and motor development, poor educational outcomes, reduced work capacity, and lower adult economic productivity (4, 6–11). Among the poorest communities, particularly in rural areas, malnutrition is compounded by socioeconomic constraints, forcing families to rely on cheaper, ultra-processed foods at an increasingly early age. This shift in dietary habits, influenced by factors such as population growth, fluctuating food prices, political instability, and environmental changes, places further pressure on the global food system and intensifies the complexity of addressing malnutrition (12–16).

To effectively combat child malnutrition, both nutrition-specific interventions, such as supplementation, and broader strategies, including agricultural improvements and social safety nets, are necessary (17–19). These methods not only address immediate causes, like nutrient deficiencies, but also tackle underlying issues related to income and food access. Forest ecosystems, for example, play a vital role in food security, particularly in rural LMICs, where forest-derived foods such as fruits, vegetables, meat, and fish meet a significant portion of dietary needs (20). A study of 24 countries found that more than 55% of rural households with moderate access to forest resources obtain food from the forest (20). Another study in Cameroon found that women in rural forest-dependent communities obtained 93% of their daily vitamin A from forest food (21).

In addition to providing direct food sources, forests also serve as important sources of income through the sale of timber and non-timber products. Research, including a review by Vedeld et al. of 51 case studies, indicates that forest products account for about 22% of these households' total income (22, 23). This additional income is frequently reinvested in food security and health, helping to safeguard nutrition and prevent nutrition-related diseases (23, 24).

Building on this foundation, Food Forests offer a promising intervention by combining ecological sustainability with agricultural productivity. These diverse, multi-layered systems go beyond traditional forestry, providing food, livelihood opportunities, and environmental benefits (25–29). Evidence from a case study in Sangthong district, Laos, illustrates the potential of Food Forests, where even less affluent families significantly improved their dietary quality through integrated agroforestry systems (30). Despite these promising indicators, however, there is a notable gap in the literature, as few comprehensive studies have rigorously quantified the impact of Food Forest interventions on nutritional, economic, and health outcomes, with most existing research being qualitative or retrospective in nature (31, 32).

The present study aims to address this gap by evaluating the impact of a Food Forest initiative on the nutritional status, socioeconomic conditions, and health outcomes of rural communities in the Colonche Parish of Santa Elena Province, Ecuador. Using a randomized, controlled trial design, this research will provide empirical evidence on the effectiveness of Food Forests as a solution to malnutrition and poverty. The findings from this study have the potential to inform similar initiatives in rural settings across Latin America and other LMICs, fostering socioeconomic development and environmental sustainability.

## 2 Objectives

### 2.1 Main objective

To evaluate the impact of the Food Forest initiative on nutritional status, socioeconomic conditions, and health outcomes in rural communities in a developing country, using the Colonche Parish, Santa Elena Province, Ecuador, as a case study.

### 2.2 Specific objectives

- To determine the effect of the Food Forest initiative on dietary diversity, malnutrition rates, and overall nutritional status of participating communities.
- To analyze changes in household income levels, employment opportunities, and economic stability as a result of the Food Forest activities.
- To examine the influence of the Food Forest initiative on physical and mental health, including general health perception, physical functionality, and emotional wellbeing among participants.

## 3 Methods

### 3.1 Study design

**Figure 1:**
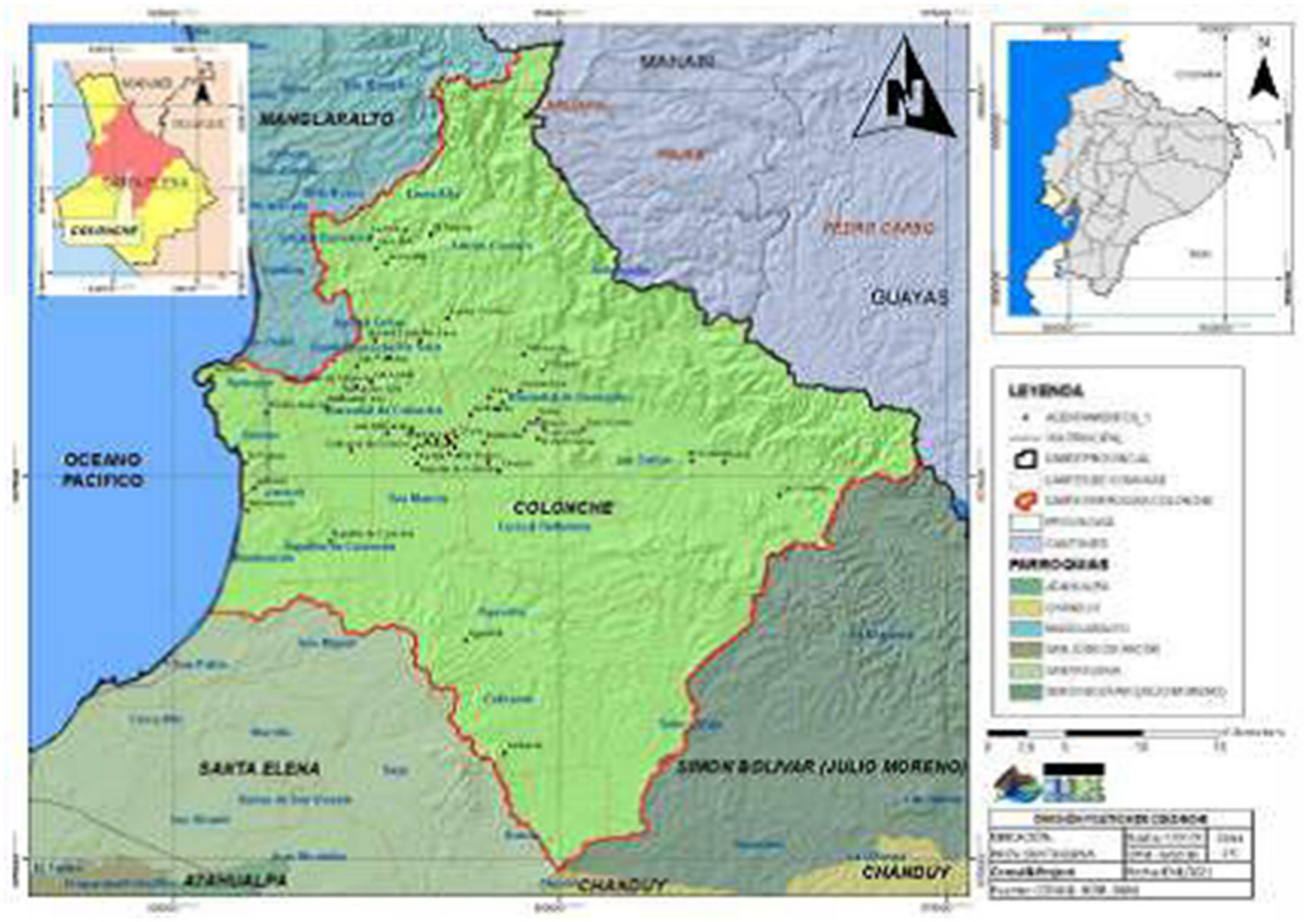
Political division of the Colonche Parish. Source: Rural Parochial Decentralized Autonomous Government of Colonche (34).

This study employs a randomized community trial design to assess the impact of a novel Food Forest reforestation technique that emphasizes the use of endemic and food-producing species. The goal is to enhance food sovereignty among coastal families in the Santa Elena Province, Ecuador. This initiative, a collaborative effort by Nedetel and CECAPROF Corporación Ecuatoriana De Capacitación (two companies that serve the area), aims to address the dual challenges of malnutrition and low average family incomes in the region (33). Participants for the study will be recruited from rural areas within the Colonche Parish of the Santa Elena Canton, a geographical area detailed in Figure 1 (34, 35). The study randomly allocated one commune to receive the Food Forest intervention, while a similarly characterized commune was randomly assigned as a control group, receiving no intervention. This randomization process ensures that the study meets the criteria of a randomized community trial. In consideration of ethical concerns, we have planned a delayed intervention for the control group. After the study period, the control commune will also receive the food forest intervention, contingent on securing additional funding. This phased approach allows for a scientifically rigorous assessment of the intervention's effects while ensuring that both communities ultimately benefit from the food forest initiative. The intervention strategy was developed based on insights from prior research, supplemented by consultations and interviews with experts in nutrition and reforestation, ensuring that the Food Forest initiative is ecologically viable and tailored to meet the nutritional and economic needs of the local communities involved.

### 3.2 Participants and recruitment

The focus of this study is on the residents of a specific commune within the Colonche Parish, located in the Santa Elena province of Ecuador. The participant pool consists of families with an average monthly income of $463.93, a figure significantly lower than the cost of the Ecuadorian basic necessities basket, estimated at $789.57 (36, 37). The predominant educational attainment among community members is up to the primary level, facilitated by the region's publicly funded educational system. Each commune is home to ~3,875 individuals, distributed across 968 households. In addressing the needs of this community, the study aims to provide direct access to food for all participating families, leveraging the coastal commune's resources to enhance their food security and nutritional wellbeing.

### 3.3 Sampling

In our study within Colonche Parish, we will engage in a randomized selection process to choose one commune for the treatment group, where the Nutritional Forest will be implemented, and two communes for the control group, which will not have access to the Nutritional Forest. This selection process will encompass all communes of the Parish, including Aguadita, Ayangue, Bajadita de Colonche, Bambil Collao, Bambil Desecho, Calicanto, Cerezal Bellavista, Febres Cordero, Jambelí, Las Balsas, Loma Alta, Manantial de Guangala, Manantial de Colonche, Monteverde, Palmar, Río Seco, Salanguillo, and San Marcos. Each commune will be treated as an individual cluster, and a random cluster sampling method will be employed to ensure a fair and unbiased selection of the communes for both the treatment and control groups.

The random selection will be carried out using a lottery system where each commune will be assigned a unique identifier, and a computerized random number generator will select the treatment commune and control communes. This approach minimizes selection bias and ensures a randomized, equitable process.

The 1:2 allocation ratio (one treatment commune and two control communes) was chosen due to feasibility considerations and community input. The local community expressed a preference for starting the Nutritional Forest intervention in one commune initially, allowing for more focused implementation and monitoring. This also aligns with the available resources and ensures that the intervention can be tailored to the specific needs of the treatment community.

Following the selection of the commune, we will proceed to sample households within each of the chosen commune. Household selection will be conducted using a two-stage sampling method. First, a list of households within each commune will be obtained from local community records. Second, a systematic sampling method will be applied to randomly select households from this list, ensuring that the sample is representative of the entire community. This step will ensure that our assessment captures a broad spectrum of community experiences and perspectives regarding the impact of the Nutritional Forest intervention.

### 3.4 Sample size

A total of 968 families were considered as the research units in this study. Therefore, considering a margin of error of 5% and a confidence level of 95%, the estimated sample size before considering the finite population correction was approximately 384, according to Equation 1 where n is the sample size, Z is the Z-score associated (1.96) with the desired confidence level (95%), p is the estimated proportion of the attribute present in the population (0.5), and E is the margin of error (5%). After applying finite population correction, the estimated sample size was reduced to 276. This adjustment is made because the population is relatively small, and the correction helps avoid overestimating the required sample size. The sample of families in each commune is 300 because attrition is expected. The heads of the household will be surveyed regarding the nutritional, socioeconomic, and health situation of the family. The primary sampling unit for this study is the household (family unit), with a total sample size of 300 families. Within each family, specific questionnaires will be administered based on the nature of the assessment. The *Household Food Insecurity Access Scale (HFIAS)* will be completed by the head of the household to capture food security at the family level. For health and quality of life assessments, individual adult members will complete the *Short Form-36 Health Survey (SF-36)* and the *World Health Organization Quality of Life (WHOQOL-100)* questionnaire to evaluate personal health and quality of life outcomes. Additionally, the *Pediatric Quality of Life Inventory (PedsQL)* will be administered to caregivers of children to assess child health-related quality of life. This approach ensures comprehensive measurement across family and individual levels, providing both household-level insights and individual health metrics.

Formula for sample size estimation.


(1) $$\begin{array}{l} n=\frac{Z^{2}\times p\times \left(1-p\right)}{E^{2}} \end{array}$$

### 3.5 Questionnaires

Data collection tools will target both family-level and individual-level outcomes. The family unit will be assessed for food security and socioeconomic factors, while individual-level data will be gathered for specific health and nutritional outcomes. For example, the *Household Food Insecurity Access Scale (HFIAS)* is designed to capture food security at the household level, while the *Short Form-36 Health Survey (SF-36)* and the *World Health Organization Quality of Life (WHOQOL-100)* questionnaire are individually administered to adult members to evaluate personal health and quality of life. Additionally, the *Pediatric Quality of Life Inventory (PedsQL)* will be used for children's health-related quality of life, completed by their caregivers.

#### 3.5.1 Nutritional situation

Nutritional status will be assessed using the *Mini Nutritional Assessment* (*MNA*) (38). The MNA is a comprehensive tool designed to evaluate nutritional status. It encompasses a series of questions and assessments that collectively provide insights into an individual's dietary habits, health status, and overall risk of malnutrition. This study will incorporate 14 specific questions from the MNA, covering various dimensions such as dietary diversity, protein intake, fruit and vegetable consumption, and any recent changes in food intake due to loss of appetite, digestive problems, or chewing issues.

Given that the *Mini Nutritional Assessment (MNA)* is intended for evaluating the risk of undernutrition specifically in older adults, this tool will be used only with older adult participants in our sample. For other age groups, nutritional status will be assessed using anthropometric measurements, including weight, height, and age, to calculate Body Mass Index (BMI) and other relevant indicators. Additionally, dietary diversity at the family level will be evaluated using a modified dietary diversity questionnaire, which is more applicable to all age groups and provides insights into household dietary patterns.

#### 3.5.2 Socioeconomic situation

The assessment of the socioeconomic situation within this study will be conducted using an adapted version of the Socioeconomic Level Stratification Survey questionnaire, originally implemented by the Instituto Nacional de Estadística y Censos (INEC) of Ecuador in 2011 (39). This comprehensive tool consists of twenty-five carefully selected questions for the Ecuadorian context designed to capture a wide range of socioeconomic indicators.

#### 3.5.3 Health situation

Health status was assessed using four questionnaires. These questionnaires were *the Short Form*-*36* Health Survey (*SF*-*36*) (40), *the Household Food Insecurity Access Scale* (*HFIAS*), *The World Health Organization Quality of Life* (*WHOQOL-100*) for adults (41), and *the Pediatric Quality of Life Inventory* (*PedsQL*) for children (42). These will include thirty-six, nine, hundred, and twenty-three questions, respectively.

To guarantee precision and contextual relevance for the Ecuadorian setting, we will employ Spanish-validated versions of all assessment tools.

#### 3.5.4 Active participation

To assess the level of engagement with the Food Forest initiative, our study will utilize a specialized self-administered questionnaire composed of five questions specifically tailored to this context. These questions are designed to explore various dimensions of participation and involvement with the Food Forest:

1. Frequency of interaction with the Food Forest: This question measures how often community members engage with the Food Forest, whether daily, weekly, or on a less frequent basis.
2. Types of activities undertaken: Participants will be asked about their involvement in specific activities such as planting, harvesting, and maintenance of the forest.
3. Contributions to planning and development: This question investigates whether participants have contributed to the planning, decision-making, or overall development of the Food Forest initiative.
4. Collaboration with other community members: Participants will report on their interactions and collaborative efforts with other community members within the Food Forest setting.
5. Perceived impact of participation: Finally, participants will be asked to assess how their involvement in the Food Forest has impacted both the forest and their personal or household well-being, covering both social and economic dimensions.

This questionnaire will provide valuable insights into how actively community members engage with the Food Forest and in what specific ways, helping to shed light on the initiative's integration into the community's social and economic fabric.

### 3.6 Data collection

Information will be collected in the homes of members of the communes. The data will be entered offline via tablets into Qualtrics (43).

### 3.7 Data management

Data entry for this research will be strictly managed by our team, ensuring access is limited to key researchers and statisticians who won't know participant identities. This safeguarding of data underscores our commitment to privacy and the secure use of information for analyzing the intervention's impact and authoring related papers.

### 3.8 Measurement slots

Participants will be assessed at baseline, 1 year, 3 years, and 5 years after the intervention starts. Their nutritional, socioeconomic, and health statuses will be assessed at each time point. Participants will also be asked about their active participation in the Food Forest Project.

### 3.9 Measurements

Primary and secondary outcome measures will be assessed in three-time points.

The primary and secondary outcomes of this study are centered around assessing the nutritional, socioeconomic, and health statuses of the participants.

#### 3.9.1 Primary outcome

Nutritional Status—The focus is on evaluating participants' nutritional health through a detailed questionnaire designed to provide both quantitative and qualitative insights into their dietary habits and the quality of their nutrition.

#### 3.9.2 Secondary outcomes

- **Socioeconomic status:** a specialized questionnaire will categorize each family's socioeconomic level into one of five strata, providing a nuanced understanding of the economic conditions influencing participants' lives.
- **Health status:** participants' overall health will be assessed using four distinct questionnaires, covering a broad spectrum of indicators including physical functionality, pain levels, general health perception, vitality, social and emotional roles, and mental health. This comprehensive approach aims to capture the multifaceted aspects of health influenced by the intervention.

### 3.10 Statistical analysis

All statistical analyses will be performed using Stata 18.0 (44). Descriptive statistics will be calculated for both qualitative (e.g., categorical) and quantitative (e.g., continuous) data. Categorical variables, such as employment status or food security, will be summarized using frequencies and percentages, while continuous variables, such as household income or nutritional status (e.g., BMI), will be described using means and standard deviations for normally distributed data, or medians and interquartile ranges for non-normally distributed data.

For hypothesis testing, categorical variables like food security (measured as secure/insecure) and employment status (e.g., employed/unemployed) will be compared between the intervention and control groups using Chi-square tests or Fisher's exact tests, depending on sample sizes. For continuous variables, such as changes in household income or nutritional status (e.g., BMI, dietary diversity scores), Student's *t*-tests will be used if the data are normally distributed. If normality assumptions are violated, Mann-Whitney U tests will be applied instead.

To evaluate changes over time within the same group (e.g., baseline to 6 months), paired *t*-tests will be used for normally distributed continuous variables, such as income or BMI, while Wilcoxon signed-rank tests will be used for non-normally distributed variables. For categorical variables like food security status over time, McNemar's test will be applied.

Regarding regression models, linear regression will be used to assess continuous outcomes such as household income levels, nutritional status (BMI), or dietary diversity scores. Logistic regression will be applied to binary outcomes, such as food security (secure/insecure) or whether participants report improved health (yes/no). For outcomes with multiple categories, such as levels of health perception (good, fair, poor) or employment status (employed, unemployed, seeking work), multinomial logistic regression will be used.

To account for repeated measures over time (e.g., baseline, 6 months, and 12 months post-intervention), mixed-effects models (both linear and logistic) will be employed. These models will allow us to assess the effect of the intervention across different time points while controlling for intra-individual correlations. All hypothesis tests will be two-tailed, and a *p* < 0.05 will be considered statistically significant.

To align with the nature of the data, nutritional and health status variables will be analyzed at the individual level, allowing us to capture specific health outcomes for each family member. Socioeconomic variables, including food security (measured by the Household Food Insecurity Access Scale [HFIAS]) and dietary diversity, will be evaluated at the family level. This approach enables a comprehensive analysis by distinguishing between individual health measures and collective socioeconomic conditions.

For older adult participants, the *Mini Nutritional Assessment (MNA)* will be used to assess undernutrition risk. For all other participants, individual nutritional status will be determined through anthropometric measurements (weight, height, and age). Household-level dietary diversity will be measured with a dietary diversity questionnaire, enabling us to capture a broader picture of household nutritional intake.

### 3.11 Ethics and dissemination

The Kennedy Clinic's Institutional Review Board (IRB) in Guayaquil has granted approval for this study, under the code HCK-CEISH-2022-006. At every phase of the research, informed consent will be rigorously obtained and mandated, adhering to the ethical standards for research involving human participants. To ethically support the control group during the study, we will provide alternative community support measures, such as nutritional education and health assessments, to ensure both communities receive beneficial resources. Additionally, we have planned a delayed food forest intervention for the control group as soon as funding allows, ensuring that both communities ultimately benefit from the initiative. This approach has been designed in collaboration with community leaders to align with ethical standards and community expectations.

## 4 Interventions and procedures

### 4.1 Development of intervention

The procedures of the intervention were developed through the consultation of experts linked to the two organizations, together with the planning of the members of the organizations themselves, in such a way that previous experiences related to the subject have been considered together with a literary review. However, the development objectives were considered in terms of economic, environmental, and health themes.

Community involvement is central to both the establishment and maintenance of the food forest. The land designated for the food forest is community-owned, allowing the benefits of the forest to be shared among all members. To ensure long-term sustainability, community members will be actively engaged in both planting and maintaining the forest. Training in agroecology and sustainable practices will be provided by CECAPROF and Nedetel, who will supply the necessary seeds and inputs, such as organic fertilizers and tools. Additionally, regular workshops will be conducted to educate the community on agroecological methods, fostering a collaborative environment where community members collectively manage the food forest, including planning, harvesting, and distribution of yields.

### 4.2 Content of intervention

The community's role in establishing and maintaining the food forest is structured through active participation in key phases of the project. Community members are involved from the onset in activities such as site preparation, planting, and periodic maintenance. Harvests are organized so that community members can collectively decide on usage, ensuring fair distribution and communal benefit. Regular meetings will also allow the community to provide input on project development, further strengthening local engagement and commitment to long-term maintenance.

The intervention comprises two key activities (plantations and reforestation workshops), conceived and implemented by Nedetel and CECAPROF, within the territories of the Colonche Parish communes. Designed to run concurrently, these activities span in total 5 years, allowing for a synchronized approach. This strategic implementation aims to address malnutrition and enhance the average family income in Santa Elena Province, contributing to overall community wellbeing.

#### 4.2.1 Plantations

The initial phase of the intervention involves an innovative reforestation effort, utilizing the Food Forest technique to plant 12,500 trees across 15 hectares within Colonche Parish. This diverse selection includes ancestral trees and various species that yield food or sustainable products. A strategic allocation ensures that 30% of these trees directly contribute to enhancing the commune's nutritional needs, while the remaining 70% are designated for commercial purposes, thereby supporting economic growth.

CECAPROF takes the lead in this endeavor, overseeing the meticulous treatment of seeds and the careful transplantation of saplings, with a commitment to achieving a 90% survival rate for the newly planted flora. Spanning ~9 months, this activity not only focuses on the technical aspects of reforestation but also establishes a reliable supply chain. A collection center, operated by CECAPROF, facilitates regular access to the harvest every 3 months, ensuring the community benefits directly from the bounty.

Moreover, this initiative is protected from external and internal interference, allowing the commune exclusive rights to utilize and benefit from the reforested land. The incorporation of georeferencing technology further enhances oversight, enabling precise monitoring of the reforestation area's growth and development, thereby ensuring the project's success and sustainability.

#### 4.2.2 Reforestation workshops

Concurrently, CECAPROF will host a series of Reforestation Workshops aimed at empowering the local community with the knowledge and skills needed to nurture and sustain the newly reforested areas. These workshops will cover a comprehensive curriculum, including seed collection techniques, seed processing methods, the construction of nurseries, and the intricacies of reforestation mapping. Additionally, participants will learn about the design and planning of reforestation projects, coupled with environmental education that highlights the significance and history of native tree species. Scheduled over 10 months, these workshops are designed to foster a deep connection between the community and their environment, ensuring the long-term success and stewardship of the reforestation efforts.

### 4.3 Potential pitfalls and unintended effects

Several potential pitfalls and unintended effects could arise during the implementation and evaluation of the Food Forest initiative. One key challenge is the variability in participant engagement. Some community members may be more actively involved in the initiative (e.g., planting, maintaining, or harvesting), while others may not fully engage, leading to differences in outcomes that may not directly reflect the intervention's effectiveness. Additionally, external factors, such as adverse weather conditions, could impact the growth of the Food Forest and, consequently, its effects on nutritional outcomes. Furthermore, there may be difficulties in retaining participants for follow-up assessments over the twelve-month study period, particularly given the rural nature of the setting, which may affect the continuity of data collection. To mitigate these risks, we will implement regular check-ins with participants, provide incentives to encourage continued engagement, and monitor environmental conditions closely to assess their impact on the project. By anticipating these challenges, we aim to minimize their potential effects on the study's results.
